# Knowledge, Attitude and Practice of Biomedical Waste Management Among Doctors and Nurses During the COVID-19 Pandemic in Puducherry: A Cross-Sectional Study

**DOI:** 10.7759/cureus.51290

**Published:** 2023-12-29

**Authors:** Pravinraj S, Darshana Zala, Janakiram M

**Affiliations:** 1 Community Medicine, Sri Lakshmi Narayana Institute of Medical Sciences, Puducherry, IND; 2 Community Medicine, Government Vellore Medical College, Vellore, IND

**Keywords:** healthcare workers, practice, attitude, knowledge, nurse, doctors, biomedical waste, covid-19

## Abstract

Background

Biomedical waste (BMW) management is an important practice that has to be followed by all healthcare workers (HCW) in health centres. COVID-19 had become a global threat, and the spread of the infection had increased drastically. Healthcare workers were already involved in managing COVID-19 patients. It is essential to have adequate knowledge, attitude and good practices among healthcare workers during the COVID-19 period. Hence, the present study was conducted to assess the knowledge, attitude and practices of COVID-19 biomedical waste management among doctors and nurses working in a private tertiary care centre in Puducherry.

Methods

A cross-sectional study was conducted to assess the knowledge, attitude and practice of BMW management among doctors and nurses who were working during the COVID-19 period. A total of 384 samples were recruited, and the study was conducted for a period of four months. Data were collected from a pre-validated, pre-tested questionnaire that assessed the knowledge, attitude and practices of biomedical waste management. Further analysis was done using Microsoft (MS) Excel (Microsoft^®^ Corp., Redmond, WA) and the Statistical Package for Social Sciences (SPSS) software (IBM SPSS Statistics, Armonk, NY).

Results

Among the 384 participants, 152 (39.6%) had excellent knowledge, 143 (37.2%) had good knowledge and 89 (23.2%) had poor knowledge. Among the 111 doctors, 55 (49.5%) had excellent knowledge, and 36 (32.4%) had good knowledge. Among the 273 nurses, 107 (39.2%) had good knowledge and 97 (35.5%) had excellent knowledge. Of the study participants, 98.2% had a favourable attitude, and 89.6% had favourable practices towards biomedical waste management. Occupation and training received on BMW management were found to be predictors of knowledge regarding biomedical waste management.

Conclusion

From the present study, it is found that the knowledge of biomedical waste management needs to be improved among doctors and nurses, especially in situations such as the COVID-19 pandemic. In this study, a positive correlation was found between knowledge score and practice score, which states that increasing knowledge regarding biomedical waste management will improve practice towards biomedical waste management. Also, doctors and nurses who had already received training in biomedical waste management were found to have better knowledge than those who had not. Hence, the college administration should do various health education activities and training sessions to enhance biomedical waste management among doctors and nurses working in the hospital.

## Introduction

The novel coronavirus has been a global threat, especially in developing countries. Countries such as India had faced a sudden spread of infection and an increased number of deaths. Healthcare workers (HCW) also had to encounter a lot of COVID-19-infected individuals. They had to deal with infectious materials that had been taken from the COVID-19 patients.

Data collected by the Central Pollution Control Board (CPCB) revealed that the country has generated 45,954 metric tonnes of 'COVID-19' waste from May 1, 2020, to May 10, 2021, which shows a 20% increase in biomedical waste (BMW) production in India [1]. Though there were rules regarding biomedical waste management [2], the knowledge regarding the disposal of biomedical waste is not clearly known to the ground-level workers, especially regarding waste generated during the COVID-19 pandemic. This led to the Central Pollution Control Board categorising it as hazardous biomedical waste as soon as the pandemic tightened its grip globally. The CPCB releases guidelines from time to time on the matter and reviews them to ensure that COVID-19 waste is collected with caution and transported in a safe manner by following all the regulations for disposal [3].

According to the Bio-Medical Waste Management Rules, 2016, 'biomedical waste' means any waste that is generated during the diagnosis, treatment or immunisation of human beings or animals or research activities pertaining thereto or in the production or testing of biological products or in health camps, including the categories mentioned in Schedule I appended to these rules [4].

Studies from various parts of India show that the majority of the biomedical wastes (75%-90%) produced by the healthcare sector are non-risky for the general public. The remaining 10%-25% of healthcare waste causes various types of health hazards to the public [2].

Biomedical waste collection, proper disposal and management have always been of significant concern for both hospital and public health settings. Since the start of the COVID-19 pandemic, disposal has taken another serious turn, as any breach in handling can wreak havoc on the global public. Disinfecting and sanitising and, more importantly, disposing of the used personal protective equipment (PPE) kits and fumigating the wards and hospitals every few hours have reduced the spread of the deadly disease to some extent.

There is a particular concern with diseases such as HIV/AIDS and hepatitis B and C, which have strongly been linked to COVID-19, as they are considered immunocompromised states, which can contribute to high mortality among COVID-19 cases [5]. The spread of the above-mentioned diseases, such as HIV/AIDS and hepatitis, is mainly blood-borne and can be transmitted from the affected patients via used syringes, cuts via used blades, blood spills and blood transfusion.

As there is a rise in different variants of COVID-19 [6], considering omicron spread, there is a need to assess the knowledge and practices of healthcare workers regarding COVID-19 waste management. A study conducted in 2012 revealed that nearly one-third of medical doctors and nurses had inadequate knowledge, and about half of medical doctors (44.0%) had poor practices in biomedical waste management [7]. It is found that knowledge, attitude and practice of biomedical waste management play a significant role in better waste management [8].

In this regard, the present study was aimed at assessing the knowledge, attitude and practices of COVID-19 biomedical waste management among doctors and nurses working in a private tertiary care centre in Puducherry.

## Materials and methods

A cross-sectional study was conducted among doctors and nurses working in a private tertiary care hospital in Puducherry. The study was conducted for a period of four months (from April to July 2021). Medical interns, resident doctors, assistant professors, associate professors, professors and nursing staff were included in the study. All the above-mentioned healthcare workers should have a minimum of six months of working experience in their respective fields. The participants who did not give consent were excluded from the study.

Sample size calculation

Assuming that the knowledge among healthcare workers will be 50% with a 95% confidence interval and 5% error, the sample size obtained was 384. The sample size was calculated using OpenEpi 3.01 (Emory University, Atlanta, GA) [9].

Sampling method

There were 891 doctors and nurses working; among them, 29% were doctors, and 71% were nurses. The total sample size needed for the study was 384. Therefore, 111 (29%) doctors and 273 (71%) nurses were included in this study using line lists obtained from the college administration, and sample selection from each stratum was done by a simple random technique.

Data collection

After explaining in detail about the study, consent was obtained from doctors and nurses. Data were collected in a pre-tested, validated, self-administered questionnaire that was made based on COVID-19 biomedical waste management and the Central Pollution Control Board [3]. The questionnaire was validated by the professors from the microbiology and community medicine departments. The tool was made in English, and a pilot study was done among three doctors and seven nurses. The reliability of all domains was tested with Cronbach's alpha (r = 0.84).

The questionnaire had four parts. The first part contains a sociodemographic profile including age, gender, year of experience, occupation and training received in biomedical waste management. The second, third and fourth parts assess knowledge, attitude and practice, respectively.

Knowledge

There were 15 questions, and one mark was given for each correct answer. After adding all the marks, analysis was done. The participants who had scores of 0-8 (less than 50%) were considered to have 'poor knowledge', 9-11 (50%-75%) as 'good knowledge' and 12-15 (more than 75%) as 'excellent knowledge'. A study conducted by Jalal et al. [10] had a similar method of collecting knowledge scores. Taking the above-mentioned study as a reference, after validating with subject experts, the present scoring system is used to collect data.

Attitude and Practice

Both had 10 questions each, and the 'yes' or 'no' response was noted according to the questions asked in each domain. Among 10 questions, the participants who gave a favourable response to more than five questions (>50%) were considered to have a favourable practice and attitude.

Data analysis

Data analysis was done with Microsoft (MS) Excel 2019 (Microsoft® Corp., Redmond, WA) and the Statistical Package for Social Sciences (SPSS) version 21.0 (IBM SPSS Statistics, Armonk, NY). The data were collected and entered in MS Excel. Frequency tables were made, and the chi-square test was used to find the association between knowledge level and other variables. Ordinal regression was performed between the knowledge level and selected variables. Pearson correlation was performed to check the relation between knowledge score and practice score of BMW management.

## Results

Table 1 shows the distribution of the study participants. Out of the 384 participants, 125 (32.6%) belonged to the age group of 26-30 years of age, and 87 (22.7%) belonged to the age group of 31-35 years of age. The majority of the study participants were female (294, 76.6%). Two hundred sixty-three (68.5%) study participants had work experience of 1-5 years, and 65 (16.9%) had 6-10 years. With respect to their occupational status, 273 (71.0%) nurses and 111 (29.0%) doctors were recruited in the study. Three hundred nineteen (83.1%) study participants had already received training in biomedical waste management before the start of the study.

**Table 1:** Sociodemographic profile BMW: biomedical waste

Variables	n (%)
Age (in years)	
21-25	69 (18)
26-30	125 (32.6)
31-35	87 (22.7)
36-40	75 (19.5)
41-45	28 (7.3)
Sex	
Male	90 (23.4)
Female	294 (76.6)
Work experience (in years)	
<1	14 (3.6)
1-5	263 (68.5)
6-10	65 (16.9)
>10	42 (10.9)
Occupation	
Doctor	111 (29.0)
Nurse	273 (71.0)
Training received on BMW management	
Yes	319 (83.1)
No	65 (16.9)

Knowledge level of the doctors and nurses on BMW management

The responses to questions asked regarding knowledge are given in the Table 2. There were a total of 15 questions asked, and the proportion of participants who gave correct answers to each question is given in Table 2.

**Table 2:** Knowledge of biomedical waste management among doctors and nurses

Knowledge-based questions	Correct answer, n (%)	Wrong answer, n (%)
Is there any rule or act available for biomedical waste (BMW) management?	310 (80.7)	74 (19.3)
Is colour coding available for BMW?	382 (99.5)	2 (0.5)
Disposal of anatomical waste has to be into which coloured bag?	337 (87.8)	47 (12.2)
Sharps disposal has to be in which coloured bag?	261 (68)	123 (32)
Up to what level does the bag have to be filled?	277 (72.1)	107 (27.9)
Personal protective equipment (PPE) includes all except?	301 (78.4)	83 (21.6)
What is the percentage of infectious waste in hospitals?	193 (50.3)	191 (49.7)
Is it necessary to have a biohazard symbol on the BMW bag?	378 (98.4)	6 (1.6)
Biomedical waste generated from quarantine centres and camps should be collected separately in which bag?	278 (72.4)	106 (27.6)
What is the method of disposal for the yellow bag?	212 (55.2)	172 (44.8)
Masks and gloves used by people other than COVID-19 patients should be kept in a paper bag for a minimum of how many hours?	166 (43.2)	218 (56.8)
PPEs such as goggles, face shields and splash-proof aprons collected during the collection of COVID-19 waste should be disposed of in which colour bag?	307 (79.9)	77 (20.1)
Solid waste bags and bins should be disinfected with what solution?	162 (42.2)	222 (57.8)
Which of the following workers should be collecting COVID-19 patients' discarded waste?	244 (63.5)	140 (36.5)
Discarded PPE from the general public at commercial establishments should be stored in a separate bin for how many days?	143 (37.2)	241 (62.8)

**Figure 1:**
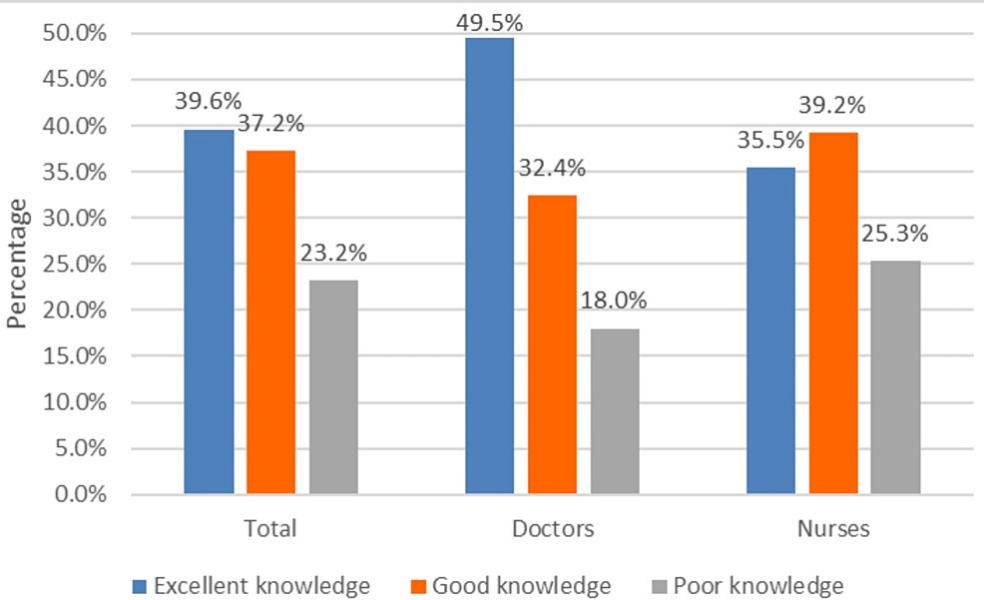
Proportion of knowledge of BMW management among doctors and nurses BMW: biomedical waste

Figure 1 shows the proportion of doctors and nurses showing the level of knowledge regarding biomedical waste management. Among 384 doctors and nurses, 152 (39.6%) had excellent knowledge, 143 (37.2%) had good knowledge and 89 (23.2%) had poor knowledge. Among doctors, 55 (49.5%) had excellent knowledge, and 36 (32.4%) had good knowledge. Among nurses, 107 (39.2%) had good knowledge, and 97 (35.5%) had excellent knowledge. The overall mean score of knowledge was 10.39 ± 2.32. Among doctors, it was 10.84 ± 2.0, and among nurses, it was 10.21 ± 2.42, and the differences were statistically significant (t = 2.441; p = 0.015).

Attitude of the doctors and nurses towards BMW management

The attitude of doctors and nurses towards BMW management was reported in Table 3. The majority of the participants, 335 (87.2%), agreed with the necessity of BMW management rules. Three hundred eighty-three (99.7%) doctors and nurses feel that BMW management is compulsorily needed for healthcare delivery. More than half of the study participants, 236 (61.5%), agreed that working in BMW management is safe. Most of the study participants, 375 (97.7%), agreed that following colour coding for waste disposal is a must. Three hundred seventy-six (97.9%) doctors and nurses agreed to advise their subordinates to follow colour coding for waste disposal. Three hundred seventy-five (97.7%) doctors and nurses believed that PPE should be worn while handling biomedical waste. The majority of the study participants, 311 (81.0%), believe that they have adequate knowledge regarding BMW management. Two hundred twenty-five (58.6%) study participants felt that there was a need for further training on BMW management. Three hundred seventy-one (96.6%) doctors and nurses agreed that segregating biomedical waste is not a difficult task. All the participants, 384 (100%), thought that taking BMW management lightly could lead to serious health hazards.

**Table 3:** Attitudes towards biomedical waste management among doctors and nurses PPE: personal protective equipment

Attitude-based questions	Yes, n (%)	No, n (%)
Is there any necessity for biomedical waste (BMW) management rules?	335 (87.2)	49 (12.8)
Do you feel that BMW management is compulsorily needed for healthcare delivery?	383 (99.7)	1 (0.3)
Do you think it is safe to work on biomedical waste management?	236 (61.5)	148 (38.5)
Do you agree that using a colour code for waste disposal is a must?	375 (97.7)	9 (2.3)
Will you advise your subordinates to follow colour coding for waste disposal?	376 (97.9)	8 (2.1)
Do you think PPE should be worn while handling biomedical waste?	375 (97.7)	9 (2.3)
Do you think your knowledge regarding BMW management is adequate?	311 (81)	73 (19)
Do you think any further training is required for BMW management?	225 (58.6)	159 (41.4)
Do you think segregating biomedical waste is not a difficult task?	371 (96.6)	13 (3.4)
Do you think BMW management, if not taken seriously, can lead to serious health hazards?	384 (100)	0 (0)

**Figure 2:**
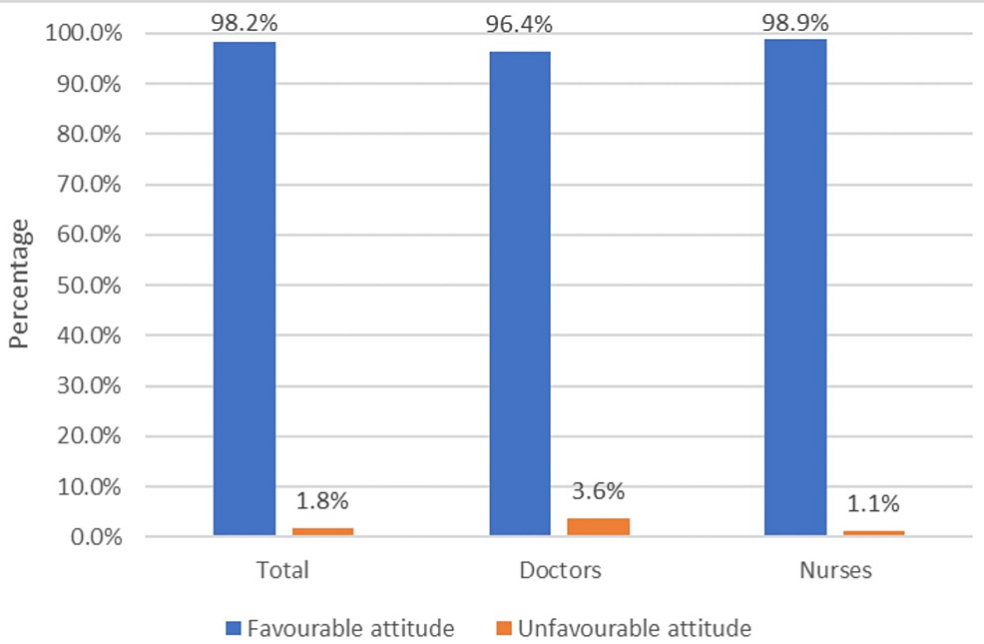
Proportion of favourable attitudes towards BMW management among doctors and nurses BMW: biomedical waste

Figure 2 shows the proportion of doctors and nurses showing a favourable attitude towards biomedical waste management. Overall, 377 (98.2%) doctors and nurses combined were showing a favourable attitude. Among doctors, 107 (96.4%) and, among nurses, 270 (98.9%) had a favourable attitude towards biomedical waste management. The overall mean scores of attitudes were 8.77 ± 0.98. The participants who gave a favourable response to more than five questions (>50%) were considered to have a favourable attitude.

Practice of the doctors and nurses towards BMW management

The practice of doctors and nurses in BMW management was reported in Table 4. Two hundred forty-one (62.8%) doctors and nurses were discarding syringes (without needles) in a red bag. Most of the study participants, 338 (88.0%), were using PPE while handling COVID-19 patients. Two hundred eighty-three (73.7%) doctors and nurses were practising hand hygiene in between every activity. Most of the study participants, 297 (77.3%), were using a sharps destructor or sharps destroyer. Three hundred seventy-nine (98.7%) doctors and nurses were using colour-coded bags for waste disposal in the COVID-19 ward. Three hundred seventy-three (97.1%) doctors and nurses informed the sanitary staff regarding BMW management once the bag was ready to be discarded. Two hundred sixty-two (68.2%) study participants were discarding expired antibiotics in a yellow bag. Three hundred sixty-three (94.5%) doctors and nurses were practising the segregation of infectious and non-infectious waste. The majority of the study participants, 347 (90.4%), were using dedicated collection bins labelled 'COVID-19' for the segregation of biomedical waste. Two hundred sixty-eight (69.8%) doctors and nurses were familiar with the guidelines prepared by the Central Pollution Control Board on COVID-19 waste management.

**Table 4:** Practice on biomedical waste management among doctors and nurses PPE: personal protective equipment

Practice-based questions	Yes, n (%)	No, n (%)
Are you discarding syringes (without needles) in a red bag?	241 (62.8)	143 (37.2)
Are you using PPE while handling COVID-19 patients?	338 (88.0)	46 (12.0)
Are you practising hand hygiene in between every activity?	283 (73.7)	101 (26.3)
Are you using a sharps destructor or sharps destroyer?	297 (77.3)	87 (22.7)
Are there colour-coded bags available for waste disposal in the COVID-19 ward?	379 (98.7)	5 (1.3)
Will you inform the sanitary staff regarding biomedical waste (BMW) management once the bag is ready to be discarded?	373 (97.1)	11 (2.9)
Are you discarding expired antibiotics in the yellow bag?	262 (68.2)	122 (31.8)
Are you practising the segregation of infectious and non-infectious waste?	363 (94.5)	21 (5.5)
Are you using dedicated collection bins labelled 'COVID-19' for the segregation of biomedical waste?	347 (90.4)	37 (9.6)
Are you familiar with the guidelines prepared by the Central Pollution Control Board on COVID-19 waste management?	268 (69.8)	116 (30.2)

**Figure 3:**
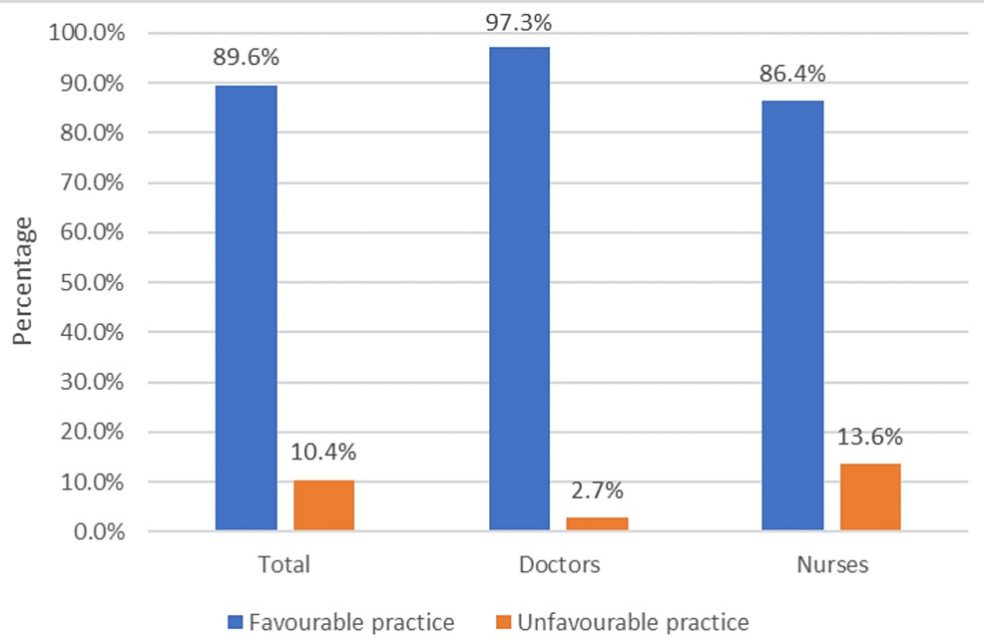
Proportion of favourable practice towards BMW management among doctors and nurses BMW: biomedical waste

Figure 3 shows the proportion of doctors and nurses showing favourable practices towards biomedical waste management. Overall, 344 (89.6%) doctors and nurses combined were showing favourable practices. Among doctors, 108 (97.3%) and, among nurses, 236 (86.4%) had favourable practices towards biomedical waste management. The overall mean scores of practices were 8.21 ± 1.61. The participants who gave a favourable response to more than five questions (>50%) were considered to have a favourable practice.

Table 5 shows the association between the level of knowledge on biomedical waste management and selected variables of the study participants. There was a statistically significant association between the level of knowledge and occupation (p = 0.035) and training received on biomedical waste management (p < 0.001).

**Table 5:** Association between the level of knowledge on biomedical waste (BMW) management and selected variables of the study participants

Variables	Poor knowledge, n (%)	Good knowledge, n (%)	Excellent knowledge, n (%)	Total (n = 384)	Chi-square (p value)
Age (in years)					
21-25	21 (30.4)	22 (31.9)	26 (37.7)	69	9.624 (0.292)
26-30	32 (25.6)	49 (39.2)	44 (35.2)	125	
31-35	21 (24.1)	28 (32.2)	38 (43.7)	87	
36-40	11 (14.7)	30 (40)	34 (45.3)	75	
41-45	4 (14.3)	14 (50)	10 (35.7)	28	
Sex					
Male	25 (27.8)	34 (37.8)	31 (34.4)	90	1.867 (0.393)
Female	64 (21.7)	109 (37.1)	121 (41.2)	294	
Work experience (in years)					
1-5	65 (24.7)	104 (39.5)	94 (35.7)	263	10.321 (0.112)
6-10	11 (16.9)	19 (29.2)	35 (53.8)	65	
<1	4 (28.6)	7 (50)	3 (21.4)	14	
>10	9 (21.4)	13 (31)	20 (47.6)	42	
Occupation					
Doctor	20 (18)	36 (32.4)	55 (49.5)	111	6.680 (0.035)
Nurse	69 (25.3)	107 (39.2)	97 (35.5)	273	
Training received on BMW management					
Yes	57 (17.8)	131 (41.1)	131 (41.1)	319	31.371 (<0.001)
No	32 (49.2)	12 (18.5)	21 (32.3)	65	
Total	89 (23.2)	143 (37.2)	152 (39.6)	384	

Table 6 shows the results of ordinal regression that was performed to determine the relationship between the levels of knowledge on biomedical waste management and selected variables of the study participants. With respect to occupation, doctors had 2.219 times the odds of an increase in knowledge level than nurses, which is statistically significant. Health workers who already received training in biomedical waste management had 3.520 times the odds of an increase in knowledge level than health workers who did not, which is statistically significant.

**Table 6:** Ordinal regression performed between the levels of knowledge regarding biomedical waste (BMW) management and selected variables of the study participants

Variables	B	Standard error	Exp(B)	95% confidence interval for Exp(B)	
				Lower bound	Upper bound
Doctors	0.797	0.226	2.219	1.425	3.456
Nurses	0				
Training received on BMW management	1.258	0.272	3.520	2.031	6.100
Training not received	0				

A Pearson correlation was performed between the knowledge score and practice score of the study participants on biomedical waste management. The Pearson correlation coefficient was 0.456 with a p value of <0.001. A positive correlation was found, which is statistically significant.

## Discussion

Biomedical waste generated during the isolation, testing and treatment of COVID-19 patients needs special consideration, as it might increase the spread of diseases among health workers if it is not treated properly. Knowledge regarding COVID-19 waste management is a must. The present study was intended to assess the knowledge, attitude and practices of COVID-19 biomedical waste management among doctors and nurses.

In this study, a total of 384 study participants were recruited; two-thirds of the participants were nurses (71.0%), and one-third were doctors (29.0%). More than two-thirds (83%) of the participants had already received training in biomedical waste management. More than two-thirds (68.5%) of the participants had working experience of 1-5 years in their respective fields. A study conducted by Jalal et al. [10] had a greater number of female participants (67.2%); nurses were more than doctors in their study. The majority (43.8%) had 1-6 years of working experience in their study. Another study conducted by Aleanizy and Alqahtani [11] also shows that more than half of the participants were female (52.5%) and more than two-thirds (74.2%) had working experience of more than five years. Makhura et al. [12] also had a majority of females (80.9%) and nurses (84.3%). A study conducted by Ashwini and Hiremath [13] on biomedical waste management among health workers had a majority of participants with experience ranging from one to 10 years. A study conducted by Dey and Das [14] found that the majority of the participants were males (64%), and work experience ranged from one to five years (54%). A study conducted in West Bengal by Dalui et al. [15] found that the majority of the participants were females (63.3%); the proportion of nurses was higher than that of doctors in their study.

Knowledge regarding biomedical waste management

In this study, among all 384 participants, more than one-third (39.6%) had excellent knowledge, more than one-third (37.2%) had good knowledge and less than one-third (23.2%) had poor knowledge regarding biomedical waste management. Among doctors, almost half of them (49.5%) had excellent knowledge, one-third (32.4%) had good knowledge and less than one-third (18.0%) had poor knowledge. Among nurses, more than one-third (39.2%) had good knowledge, more than one-third (35.5%) had excellent knowledge and less than one-third (25.3%) had poor knowledge. Jalal et al. [10] found that almost half of the participants (41.0%) had excellent knowledge, one-third (34.0%) had good knowledge and less than one-third (25.0%) had poor knowledge with respect to biomedical waste management. Dalui et al. [15] show that 43.2% had excellent knowledge, 38.5% had good knowledge and 18.2% had poor knowledge regarding biomedical waste management. Another study conducted by Makhura et al. [12] shows that almost half of them (47.2%) had adequate knowledge. More than two-thirds (77.8%) of nursing staff had good knowledge, according to the study by Ashwini and Hiremath [13]. Almost all the above studies show that one-third of the study participants have poor knowledge regarding BMW management.

In the present study, the overall mean score of knowledge was 10.39 ± 2.32. Among doctors, it was 10.84 ± 2.0, and among nurses, it was 10.21 ± 2.42. There is a slight increase in the mean score of doctors compared to nurses. Jalal et al. [10] had 20 questions in their study to assess knowledge regarding biomedical waste. In their study, the overall mean score of knowledge was 13.1 ± 3.6. Among doctors, it was 14.4 ± 3.2; among nurses, 13.6 ± 3.8 was noted. This difference is similar to the current study. Dey and Das [14] show that among resident doctors, the mean knowledge score was 8.060 ± 0.6518, and among nurses, it was 8.320 ± 0.957. Since the study was conducted on resident doctors, nurses would have had a slight increase in their knowledge mean score.

Attitude towards biomedical waste management

In the present study, among 384 participants, 98.2% had a favourable attitude towards biomedical waste management. Among doctors, 96.4% and, among nurses, 98.9% were seen. Jalal et al. [10] had 73.1% favourable attitudes. In the study by Dalui et al. [15], almost two-thirds (74.1%) had a favourable attitude. Among doctors, 91% and, among nurses, 81% had a favourable attitude towards biomedical waste management. In the study by Ashwini and Hiremath [13], 98.8% of the participants had a favourable attitude towards BMW management.

In the present study, the overall mean scores of attitudes were 8.77 ± 0.98. Dey and Das [14] show that among resident doctors, the mean attitude score was 17.84 ± 2.852, and among nurses, it was 20.78 ± 2.043.

Practices towards biomedical waste management

In the current study, among 384 participants, 89.6% had favourable practices towards biomedical waste management. Among doctors, 97.3% and, among nurses, 86.4% had favourable practices. The favourable practices among doctors on biomedical waste management were higher when compared to nurses. Makhura et al. [12] show that 61.8% of healthcare workers were following correct practices regarding biomedical waste management. Ashwini and Hiremath's [13] study shows that 87.1% of nurses had correct practices.

In the present study, the overall mean scores of practices were 8.21 ± 1.61. Dey and Das [14] show that among resident doctors, the mean practice score was 8.86 ± 1.714, and among nurses, it was 11.84 ± 1.167.

The distribution of the levels of knowledge has been significantly associated with the occupation of the study participants and with training received in biomedical waste management, which were statistically significant. The result of ordinal regression shows that occupation and training received in BMW management were found to be predictors of knowledge regarding BMW management. It should be noted that among doctors, the favourable practice towards BMW management was higher than among nurses.

There was a positive correlation between the knowledge score and the practice score of the study participants with respect to biomedical waste management. This shows that as knowledge increases, the good practice of biomedical waste management increases. A study conducted by Shekoohiyan et al. [16] also shows a strong positive correlation between knowledge and practices of biomedical waste management among healthcare workers.

Limitations of the study

The study was conducted in a single tertiary care centre in Puducherry due to the COVID-19 situation, and hence, the results obtained may not represent other tertiary care centres from different regions of the country. This study could have been done at a multicentric level for better representation. Data collection was done during the busy working hours of the study participants; there might be a chance of recall bias. Since healthcare workers already have busy working schedules, we cannot include all other healthcare workers in the study.

## Conclusions

Healthcare workers, such as doctors and nurses, were at greater risk of being exposed to COVID-19 infections. They had to work more during COVID-19 times than before. Hence, knowledge and practices regarding biomedical waste management are a must to prevent the spread of COVID-19 infections among healthcare workers and in the community. In the present study, though the majority of the people had good to excellent knowledge regarding biomedical waste management, almost one-fifth of the participants did not have adequate knowledge about biomedical waste management. Regression analysis shows that health workers who received training in biomedical waste management had better knowledge than those who did not. The Pearson correlation performed between knowledge and practice scores also shows a positive correlation between them. Hence, hospital management and the infection control committee should provide health education regarding biomedical waste management to all healthcare providers. Several activities should be conducted to increase the knowledge level. Proper training should be done on biomedical waste management, and frequent assessments should be done to check their knowledge, attitude and practices regarding waste management. It is the only key to preventing the cross-contamination of COVID-19 infections in the hospital setting.
